# Prevalence and Predictors of Depression Amongst Hypertensive Individuals in Karachi, Pakistan

**DOI:** 10.7759/cureus.1397

**Published:** 2017-06-26

**Authors:** Samar Mahmood, Syeda Z Hassan, Muqadus Tabraze, Mohammad O Khan, Iqra Javed, Ameer Ahmed, Omer M Siddiqui, Mehek Narmeen, Maham J Ahmed, Afreen Tariq, Mustafa S Patel, Kaneez Fatima

**Affiliations:** 1 Department of Internal Medicine, Dow University of Health Sciences, Karachi, Pakistan; 2 Department of Internal Medicine, Ziauddin University, Karachi, Pakistan

**Keywords:** hypertension, blood pressure, depressive, mental health, phq-9, pakistan, prevalence, associations, tertiary care, predictors

## Abstract

**Objective:**

While studies evaluating the prevalence of depression and hypertension have been extensively carried out in high income countries, there is a paucity of information assessing the prevalence of depression within hypertensive patients in low income nations. The primary objective of this study was to investigate the prevalence of undiagnosed depression in hypertensive patients within a tertiary care facility in Karachi, Pakistan. The secondary objective was to assess factors associated with undiagnosed depression in this group.

**Methods:**

A cross-sectional study was conducted at the Civil Hospital Karachi Outpatient Department from January 2017 to April 2017. The sample population was composed of 411 hypertensive patients. Interviews were conducted after taking informed consent, with data concerning basic demographic details and lifestyle habits gathered. Blood pressure was recorded and its severity was classified as per the Joint National Committee on Prevention, Detection, Evaluation, and Treatment of High Blood Pressure (JNC-7) guidelines. Depression was evaluated and its severity classified as per the Patient Health Questionnaire-9 (PHQ-9) scale, with a score of 10 or above set as the cut-off point. Data were entered and analyzed using the IBM Statistical Package for the Social Sciences 23.0. (IBM, NY, USA)

**Results:**

The prevalence of depression within 411 hypertensive patients was 40.1% (*n *= 165). The mean age of the sample was 45.7 ± 11.2 years, and the majority were females (72%, *n *= 295), unemployed (72%, *n *= 296), had primary or no education (67%, *n *= 277), and were of low socioeconomic status (78%, *n *= 321). The average systolic and diastolic blood pressures were 143.8 ± 21.7 and 93.3 ± 15.5 mm Hg, respectively. Factors which had a significant association with depression were gender (*p *= 0.009), age class (*p *= 0.035), educational status (*p *= 0.000), employment status (*p *= 0.003), socioeconomic status (*p *= 0.008), physical activity (*p *= 0.025), smoking (*p *= 0.017), and family history of hypertension (*p* = 0.022).

**Conclusion:**

With such a high prevalence rate of undiagnosed depression within hypertensive patients, it is pertinent to establish screening programs for early detection and community programs to raise awareness regarding long-term complications of untreated depression.

## Introduction

Depression affects 350 million people around the world with a lifetime risk of 7% [1]. Depression is likely to cause a 5.7% increase in the global burden of disease by 2020 and is to become the leading cause of disability worldwide by the year 2030 [1-2]. Similarly, hypertension is one of the leading causes of global mortality and disability. In 2010, it had been estimated that a 31.1% of the global population (i.e. 1.39 billion) was hypertensive [3]. Accountable for 9.4 million deaths annually, hypertension is responsible for a variety of diseases, such as cardiovascular diseases, renal failure, and stroke. Prior studies have acknowledged the link between hypertension and depression, but the results vary. Several studies corroborate the hypothesis between the associations of depression among hypertensive patients [4]. In fact, the two may be associated with each other due to increased adrenergic activity during the state of depression, which has a pressor effect on the cardiovascular system [5]. On the contrary, few studies supported the notion of decreased blood pressure in anxious or depressed patients [6], as opposed to some that found no association [7].

Pakistan has an overall prevalence of depression of 34% [8], which is greater than the 10% prevalence within the United States. Studies show that economically developing countries have a greater propensity toward hypertension [8-9]. Despite the increased prevalence of hypertension and depression within Pakistan, few studies have been conducted in lower to middle range gross domestic product countries, such as Nepal and Pakistan, to assess the relationship between the two comorbidities [9-10]. Depression and hypertension combined have a far more detrimental effect on health than individually and are reported to decrease the quality of life and cause an increased risk of myocardial infarction and stroke [11-12]. Therefore, a study focusing on comorbid hypertension and depression in Pakistan is useful as it would allow for better health care planning and a better approach toward patient risk assessment.

Considering the paucity of data, especially in our part of the world, the primary objective of this study was to assess the prevalence of undiagnosed depression in hypertensive patients in Pakistan. The secondary objective of this study was to determine suitable associations that link depression in hypertensive patients to factors such as age, gender, and employment status.

## Materials and methods

A tertiary care hospital-based cross-sectional study was conducted from January 2017 to April 2017 in Civil Hospital Karachi after approval from the institutional review board of Dow University of Health Sciences. A sample size of 196 was calculated using openepi.com; however, 450 patients were included in this study to get a better representation of the population.

The first part of our devised questionnaire consisted of demographic details for which participants were interviewed and information regarding age, sex, ethnicity, educational status, monthly income, comorbidities, family history of hypertension, family history of depression, physical activity, dietary, and lifestyle factors were collected alongside details of anti-hypertensive medication prescription and usage. The second part of the questionnaire assessed the stage/degree of hypertension according to the guidelines set out by the Joint National Committee on Prevention, Detection, Evaluation, and Treatment of High Blood Pressure (JNC-7) [13]. The last part of the questionnaire consisted of the Patient Health Questionnaire-9 (PHQ-9), a nine-item module with a score ranging from 0 to 27. The scores are divided into five classes where a score of 0–4 shows normal range or full remission, 5–9 shows minimal depressive symptoms, 10–14 shows major depression of mild severity, 15–19 shows major depression with moderate severity, and a score of 20 or higher equals major depression of severe severity. At nine items, the PHQ-9 is half the length of numerous other depression measures, has comparable sensitivity and specificity and consists of the actual nine criteria upon which the diagnosis of Diagnostic and Statistical Manual of Mental Disorders-IV depressive disorders are based [14]. The questionnaire was translated into the local language for ease of accessibility. Blood pressure readings for the study were conducted according to the strict guidelines using a mercury column sphygmomanometer. All participants were asked to refrain from consumption of food or beverages 30 minutes prior to taking the blood pressure readings. The blood pressure readings were taken in a sitting position with the patient’s arm positioned roughly at the level of their heart. An average blood pressure reading was then calculated from the two readings obtained.

Only patients between the age of 25 and 65 years and suffering from hypertension for a time period of at least six months were included in this study where hypertension was defined as having a systolic blood pressure greater than or equal to 140 mm/Hg or a diastolic blood pressure greater than or equal to 90 mm/Hg, taking anti-hypertensive medication or being diagnosed by a professional health care worker. Our exclusion criteria included patients suffering from comorbidities such as diabetes, renal disease, cancer, previous incidences of myocardial infarction or stroke, and anyone who had been diagnosed with depression and/or any other psychiatric disorders. Pregnant women were also excluded from this study.

In order to reduce bias within the study, multiple methods were utilized. The devised questionnaire was authenticated by means of two doctors reviewing it, and a pilot study was run using 20 participants to improve understanding and minimize flaws. A standard protocol of using similar translations, attire, and attitude with the sample population was adopted by the interviewers as well. Furthermore, no imputation of missing data was carried out. Recall bias was minimized by asking questions concerning daily habits of the sample population as well as assessing depressive symptoms from no earlier than two weeks prior to the time of interviewing as per the PHQ-9 standard.

Using a convenience sampling method, 450 patients were interviewed with written consent, of which 411 provided complete data that was included in the analysis yielding a response rate of 91.3%. Data were entered and analyzed using the IBM Statistical Package for the Social Sciences 23.0 (IBM, NY, USA). In our study, the cut-off score for the confirmatory state of depression was set at 10 as per the PHQ-9 scoring, in line with the recommended value of multiple studies [15]. Systolic and diastolic blood pressure readings were categorized dichotomously as greater than/equal to or lesser than 140 and 90, respectively, whereas the severity of hypertension was deduced as per the JNC-7 guidelines. Low physical activity, poor diet, smoking, and alcohol consumption were classified according to the 2012 World health Organization (WHO) guidelines [16]. Descriptive statistics and frequencies for each variable were calculated and represented as percentages. Statistical tests were run to find associations between all the variables with the state of depression (depressed or not depressed), classification of depression (none, mild, moderate, moderately severe or severe), and a total score obtained in the PHQ-9. A *p* value of less than 0.05 was considered to be significant. All dichotomous data were tested using the chi-square test, whereas non-parametric tests were used to assess other variables, owing to them not being normally distributed.

## Results

Out of the 411 hypertensive patients (Table 1), 295 (72%) were women. The mean age of the entire population was 45.7 ± 11.2 years. An overwhelming amount of the population was either unemployed (*n *= 296, 72%), married (*n *= 388, 94%), had a monthly income of less than PK Rs. 20,000, and were in the “low” category of the socioeconomic status (*n *= 321, 78%). Amongst the educational status, most of the population had received primary or no education (*n *= 277, 67%). The mean years from the time since any individual from our study was diagnosed with hypertension was 5.2 ± 6.1 years. The average systolic and diastolic blood pressures were 143.8 ± 21.7 and 93.3 ± 15.5 mm Hg, respectively, a majority of which were categorized as stage 2 of hypertension (*n *= 151, 37%).

**Table 1:** Sociodemographic and basic clinical characteristics of participants by depression status

Variable	Total	No Depression (PHQ < 10)	Depression (PHQ ≥ 10)	*P*-value
Sex				
Male	116(28%)	82(71%)	34(29%)	0.005
Female	295(72%)	164(56%)	131(44%)	
Age (Years)				
25–44	173(42%)	92(53%)	81(47%)	0.019
45–65	238(58%)	154(65%)	84(35%)	
Educational Status				
No Education or Primary	277(67%)	144(52%)	133(48%)	0.000
Secondary	79(19%)	61(77%)	18(33%)	
Undergraduate	18(4%)	11(61%)	7(39%)	
Graduate	22(5%)	15(68%)	7(32%)	
Post Graduate	15(4%)	15(100%)	0(0%)	
Marital Status				
Married	388(94%)	231(60%)	157(40%)	0.589
Unmarried	23(6%)	15(65%)	8(35%)	
Employment Status				
Employed	115(28%)	78(68%)	37(32%)	0.040
Unemployed	296(72%)	168(57%)	128(43%)	
Socioeconomic Status				
Low	321(78%)	184(57%)	137(43%)	0.048
High	90(22%)	62(69%)	28(31%)	
Average Systolic Blood Pressure (mmHg)				
<140	154(38%)	86(56%)	68(44%)	0.199
≥140	257(63%)	160(62%)	97(38%)	
Average Diastolic Blood Pressure (mmHg)				
<90	118(29%)	79(70%)	39(30%)	0.063
≥90	293(71%)	167(57%)	126(43%)	
Severity of Hypertension				
Pre-hypertensive	135(33%)	82(61%)	53(39%)	0.959
Stage 1	125(30%)	72(58%)	53(42%)	
Stage 2	151(37%)	92(61%)	59(39%)	

**Figure 1:**
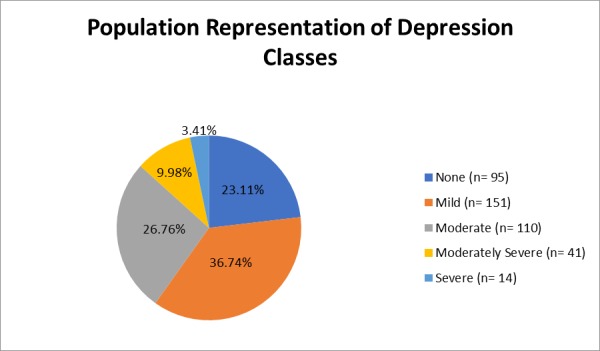
Population representation of depression classes

Overall, when using a PHQ-9 cut-off score of ≥10 for the confirmatory state of depression [15], 2/5^th^ of the population was diagnosed as depressed (40.1%, *n *= 165), women (44%) and those younger than 45 years of age (47%) more so than men (29%) and the older population (35%) (Table 1). Following the standard protocol of the PHQ-9, five classifications were made to establish the severity of depression as per which the majority was found to be mildly depressed (37%, *n *= 151), followed by those moderately depressed (27%, *n *= 110), and those in the moderately severe category (10%, *n *= 41) with the mean score being 8.62 ± 5.24 (inter-quartile range: 12–5). Only a few (*n *= 14) were severely depressed and 23% were not depressed at all (*n *= 95) (Figure 1). Statistical differences (*p *< 0.05) were observed for gender, age, educational status, employment status, socioeconomic status, and diastolic blood pressure, against the confirmatory state of depression. Apart from the female gender and the younger age group, depression was also found to be more prevalent among those with no or primary education (*n *= 133, 48%), the unemployed (*n *= 128, 43%), in the low socioeconomic status (*n *= 137, 43%), and in those with diastolic pressure >90 mm Hg (*n *= 126, 43%) (Table 1).

The health behaviors and comorbidities of participants stratified on the basis of depression status are presented in Table 2. Statistical significance (*p *< 0.05) with depression status was only seen for physical activity (*p *= 0.043) and family history of hypertension (*p *= 0.007). Participants exercising <150 minute/week had 44% prevalence rate for depression compared to those exercising ≥150 minute/week who had 33%. When asked about family history of hypertension and depression, more confirmed the former (61%) than the latter (20%).

**Table 2:** Health behaviors and comorbidities of participants by depression status

Variable	Total	No Depression (PHQ < 10)	Depression (PHQ ≥ 10)	*P*-value
Physical Activity				
<150 Minute/Week	278(68%)	157(56%)	121(44%)	0.043
≥150 Minute/Week	133(32%)	89(67%)	44(33%)	
Fruits and Green Vegetables				
<2 Serving/Day	212(52%)	120(57%)	92(43%)	0.165
≥2 Serving/Day	199(48%)	126(63%)	73(37%)	
Smoking				
Yes	244(59%)	139(57%)	105(43%)	0.149
No	167(41%)	107(64%)	60(36%)	
Heavy Episodic Drinking of Alcohol				
Yes	3(0%)	3(100%)	0(0%)	0.155
No	408(100%)	243(60%)	165(40%)	
Betel Nut Chewing				
Yes	82(20%)	56(68%)	26(32%)	
No	329(80%)	190(58%)	139(42%)	
Family History of Hypertension				
Yes	249(61%)	136(55%)	113(45%)	0.007
No	162(39%)	110(68%)	52(32%)	
Family History of Depression				
Yes	83(20%)	42(51%)	41(49%)	0.054
No	328(80%)	204(62%)	124(38%)	
Taking Medicine				
Yes	366(89%)	218(60%)	148(40%)	0.731
No	45(11%)	28(62%)	17(38%)	
Prescription for Medicine				
Yes	360(88%)	217(60%)	143(40%)	0.641
No	51(12%)	29(57%)	22(43%)	
Recommended Dose Followed				
Yes	250(61%)	153(61%)	97(39%)	0.488
No	161(39%)	93(58%)	68(42%)	

Table 3 indicates that when adjusted for total PHQ-9 score, there was a statistical difference (*p *< 0.05) observed for gender (*p *= 0.009), educational status (*p *= 0.000), employment status (*p *= 0.003), socioeconomic status (*p *= 0.008), diastolic blood pressure (*p *= 0.006), physical activity (*p *= 0.025), smoking (*p *= 0.017), family history of hypertension (*p *= 0.022), and family history of depression (*p *= 0.002). With relevance to depression class (none, mild, moderate, moderately severe or severe), a statistical difference was observed for gender (*p* = 0.026), educational status (*p *= 0.000), employment status (*p *= 0.047), socioeconomic status (*p *= 0.027), diastolic blood pressure (*p *= 0.009), physical activity (*p *= 0.016), smoking (*p *= 0.031), family history of hypertension (*p *= 0.028), and family history of depression (*p *= 0.002). In addition, depression class associations reached statistical significances with the age class (*p *= 0.035) and monthly income (*p *= 0.003) while only the total score was found to have significance with alcohol consumption (*p *= 0.049). The total score was observed to have a moderate positive correlation with monthly income and a weak negative correlation with time since hypertension was diagnosed and years since smoking.

**Table 3:** Associations with total PHQ-9 score and depression class

Variable	Total Score (*p*-value)	Depression Class (*p*-value)
Sex	0.009	0.026
Age	–	0.005
Marital Status	0.355	0.438
Educational Status	0.000	0.000
Employment Status	0.003	0.047
Monthly Income	–	0.003
Socioeconomic Status	0.008	0.027
Average Systolic Blood Pressure	0.148	0.175
Average Diastolic Blood Pressure	0.006	0.009
Time Since Hypertension Diagnosed	–	0.116
Severity of Hypertension	0.604	0.847
Dietary	0.119	0.233
Physical Activity	0.025	0.016
Smoking	0.017	0.031
Years Since Smoking	–	0.168
Alcohol Consumption	0.049	0.095
Betel Nut Chewing	0.058	0.063
Prescription for Medicine	0.902	0.858
Compliance with Medicine	0.344	0.164
Number of Medicine Taken	0.566	0.455
Family History of Hypertension	0.022	0.028
Family History of Depression	0.002	0.002

## Discussion

To our knowledge, this is the first cross-sectional study from Pakistan to evaluate the prevalence of undiagnosed depression amongst hypertensive individuals in a public, tertiary care hospital setting. Our finding of 40.1% hypertensive individuals being depressed is much higher than the 15% found in the hypertensives in a tertiary care clinic in Nepal, who had a notably higher educational status and prominently healthier lifestyle habits [9]. This finding is also higher than that observed in other groups of people in the country. For example, the prevalence of depression was 16.9% amongst the diabetics reporting to a specialist diabetes center [17], 31.8% amongst pregnant women in Lahore [18], and 34% in the general Pakistani population [8]—all lower than the prevalence in this study. However, the percentage is lower than that found amongst medical and surgical patients admitted in a tertiary care hospital in Karachi (51.2%), which also used the PHQ-9 [19]. A greater percentage of hypertensives with depression (40.1%) were found in our study, as opposed to depression patients with hypertension (21.2%) by Grimsrud, et al. in South Africa [20]—suggestive of depression being more likely to develop as a comorbid of hypertension than vice versa. The deductible reasons for this high prevalence of depression amongst hypertensive patients include the possible mental impact of being aware of having such a lifelong condition and vicious cycle of economic constraints in low socioeconomic settings, health care costs, the resultant stresses, and further disability.

We found significant associations of both the total score and the depression class of hypertension patients (both as per PHQ-9) with gender, physical activity, education, socioeconomic, and employment statuses; common between score and the confirmatory state of depression (PHQ ≥ 10) were smoking and a family history of depression. Our study backs the existent finding of depression being more prevalent in women compared to men, which has been attributed to factors, such as hormonal fluctuations, higher rates of illness, and a more severe mental burden with regards to women’s cultural role and relationships, especially in developing countries, such as Pakistan [8,21]. The association of depression with socioeconomic status reinforces how low socioeconomic backgrounds and poverty, with their associated challenges, have long been known to be predisposing factors for the condition [22]. This was proven by the participants constantly linking their depressive symptoms to their financial constraints and resulting distresses themselves. The association is further highlighted by how our finding of 40.1% depressed participants is higher than the 32.86% found amongst the residents of an elitist residential area of the city (belonging to the high socioeconomic class) [23]. Furthermore, low socioeconomic settings usually come with a mass low education status and our case was no different. A lower educational level translates to a lack of the protective effect that such mental resources grant against depression, in terms of a higher resilience to stresses [24] and more awareness of how to recognize and tackle the condition in its early stages.

Further, under personal demographics, age was the only significant association common between class (none, mild, moderate, moderately severe or severe) and a confirmatory state of depression. This finding is in agreement with the study by Barua, et al. that discussed the increasing prevalence of elderly depressives in developing countries, as opposed to other countries of the world, in light of the lack of advanced diagnostic instruments that differentiate dementia from depression, thus preventing a misdiagnosis and providing suitable mental health care and support services [25] that enable the treatment of depression at younger ages, thereby preventing its progression and controlling its intensity in the later years.

The significant association of depression with poor lifestyle habits, like smoking and a sedentary lifestyle, reinforces the need of modifying daily routines and habits in accordance with the new concept of ‘lifestyle medicine.’ Smoking is particularly a potential risk factor for the development of de-novo depression due to its involvement with the dopaminergic system and its aggravation of inflammation and oxidative stress [26]. However, an increased risk of developing major depressive disorders is influenced by more than just lifestyle factors—hereditary aspects, such as a family history of depression, have been strongly established risk factors [27]. Coupled with both the negative stigma attached with mental disorders and poor awareness of depression—familial or otherwise—in Pakistan, such an association is unsurprising.

Moreover, in relation to lifestyle habits and choices, it must also be emphasized that we did not find statistically significant associations between the taking of anti-hypertensive medication or adherence to medication and the confirmatory state, classes or score of depression. This was contrary to the existing literature that holds strong associations, both ways— in terms of decreased depression scores with the use of anti-hypertensives [9] and anti-hypertensive agents, like propranolol, being associated with depression [28] in different studies.

There are several limitations to our study. Our participants may have chosen to give socially acceptable responses instead of speaking of their symptoms honestly at times, given the method of interviewing being self-reporting, since there is still a stigma attached with mental illnesses in developing countries. Our study may overplay the prevalence of depression amongst hypertensives due to how it was conducted in just one outpatient department, with a small sample size and in a low socioeconomic setting, which is a strong predisposing factor of depressive disorders. Our tool, the PHQ-9, has been proven to have only a modest diagnostic utility in discriminating between patients with and without dementia and that may have interfered with our findings of depression in the survey population [29]. Lastly, an association between the specific type of anti-hypertensive agent being taken and the prevalence of depression could not be made.

## Conclusions

The percentage of hypertensive patients reporting to the Civil Hospital Karachi Outpatient Department found to have undiagnosed depression was extremely high. The underlying causes of depression need to be addressed and community programs need to be initiated to raise awareness regarding long-term complications of untreated depression, especially in hypertensive females. Our study also reaffirms the urgency of hypertension prevention and treatment, of educating the general population regarding the lifestyle modifications that protect against such conditions and of the need for counselling the public to have them recognize, own, and treat their depressive conditions instead of avoiding them.
